# Surgical Management of a Periprosthetic Femoral Fracture Above a Total Knee Arthroplasty: A Case Report

**DOI:** 10.7759/cureus.91445

**Published:** 2025-09-01

**Authors:** Paul Gerges, Vincent Lee, Brock Snyder, Ricardo Rios, Ralph Rizk

**Affiliations:** 1 Medicine, Edward Via College of Osteopathic Medicine-Carolinas Campus, Spartanburg, USA; 2 Medicine, Edward Via College of Osteopathic Medicine-Auburn Campus, Auburn, USA; 3 Orthopedic Surgery, Ralph Rizk Orthopedics, Jacksonville, USA

**Keywords:** bone, distal femur fracture, fixation plate, periprosthetic femur fracture, total knee arthroplasty (tka)

## Abstract

Periprosthetic distal femur fractures above total knee arthroplasty (TKA) are challenging due to compromised bone quality and the need to preserve the prosthesis. With increasing TKA volumes, the incidence of these injuries is rising. A 78-year-old female patient presented with left thigh pain and inability to bear weight following a fall. Imaging revealed a comminuted distal femur fracture proximal to a well-fixed TKA. Open reduction and internal fixation was performed using the AxSOS 3 Ti Distal Lateral Femur Plating System (Stryker, Portage, MI) via a lateral approach. Fixation was achieved without compromising the implant. The patient progressed to partial weight-bearing at six weeks and full weight-bearing by 12 weeks. At four months, she regained pain-free ambulation with radiographic union and no evidence of implant failure. This case highlights the successful use of locking plate fixation for a distal femur periprosthetic fracture. It emphasizes the importance of selecting fixation over distal femur replacement when bone stock is adequate and the prosthesis is stable.

## Introduction

Periprosthetic distal femur fractures are a growing clinical concern, particularly as the number of total knee arthroplasties (TKAs) continues to rise with an aging population. Although these fractures remain relatively uncommon, their incidence is increasing and is expected to climb further as more primary TKAs are performed in older adults with osteoporotic bone [1]. These injuries most commonly occur following low-energy trauma and pose significant treatment challenges due to compromised bone quality, limited fixation options, and the presence of an existing prosthesis [2].

Management goals include restoring limb alignment, maintaining prosthesis stability, and enabling early mobilization to minimize complications such as joint stiffness, thromboembolism, and nonunion. Selecting the appropriate surgical intervention requires careful evaluation of several factors, including fracture pattern, bone stock, patient comorbidities, and implant stability. While distal femur replacement may be indicated in cases of severe comminution or poor bone quality, locking plate fixation remains the preferred approach in patients with adequate bone stock and a well-fixed prosthesis [3].

When treating periprosthetic distal femur fractures in patients with osteopenic bone, multiple fixation strategies are available, including dual plating, combined intramedullary and plate constructs, and distal femur replacement. In this case, the patient’s fracture pattern, adequate condylar bone stock, and prosthesis stability favored single locking plate fixation, allowing stable reconstruction while minimizing surgical morbidity.

This case report describes the successful use of the AxSOS 3 Ti Distal Lateral Femur Plating System (Stryker, Portage, MI) to treat a comminuted Arbeitsgemeinschaft für Osteosynthesefragen (AO)/Orthopaedic Trauma Association (OTA) 33A fracture above a well-fixed TKA. Given the limited number of detailed operative reports in this specific context, this case contributes to the growing body of experience by outlining the rationale for implant selection, surgical technique, and mid-term follow-up. However, this approach should not be generalized, as single-case reports are inherently limited in their ability to establish definitive treatment recommendations.

## Case presentation

**Figure 1:**
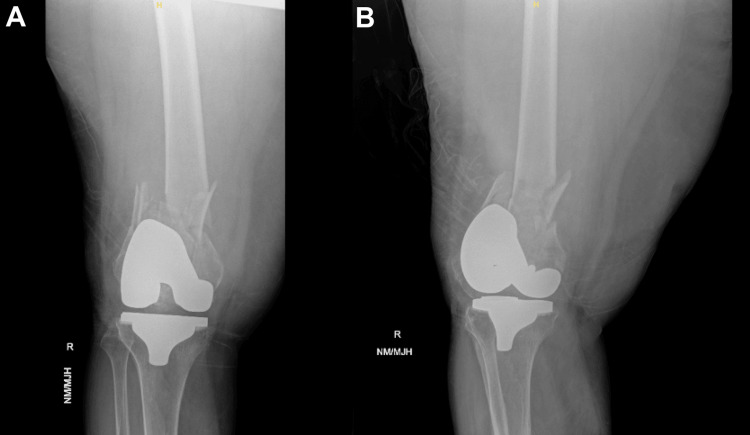
Initial radiographs of the right knee demonstrating a periprosthetic femoral fracture (A) Anteroposterior and (B) lateral radiographs of the right knee demonstrating a comminuted periprosthetic distal femoral fracture (Arbeitsgemeinschaft für Osteosynthesefragen (AO)/Orthopaedic Trauma Association (OTA) 33A3) located proximal to a well-fixed TKA implant. The fracture extends proximally into the diaphysis with displacement and fragmentation.

A 78-year-old female patient presented to the emergency department with pain and an inability to bear weight on her left leg following a fall. She had a history of TKA on the left knee five years prior, with no complications since. Clinical examination revealed tenderness and swelling over the distal femur, with no signs of neurovascular compromise. Initial imaging, as shown in Figure 1, confirmed a comminuted distal femur fracture located proximal to the TKA implant. 

Given the presence of a TKA, fixation options were evaluated carefully to avoid compromising the prosthesis while ensuring fracture stability. After multidisciplinary consultation, the AxSOS 3 Ti Distal Lateral Femur Plating System was selected for its ability to provide strong lateral fixation without interfering with the prosthesis stem.

The patient was positioned supine, and a lateral approach to the femur was performed. Careful dissection using a subvastus approach preserved surrounding soft tissues, and the AxSOS 3 Ti plate was contoured to match the distal femur’s anatomy. Multiple locking screws were applied proximally and distally, avoiding the prosthesis stem, with attention to achieving optimal alignment and fixation stability. Intraoperative radiography confirmed proper plate placement and alignment.

**Figure 2:**
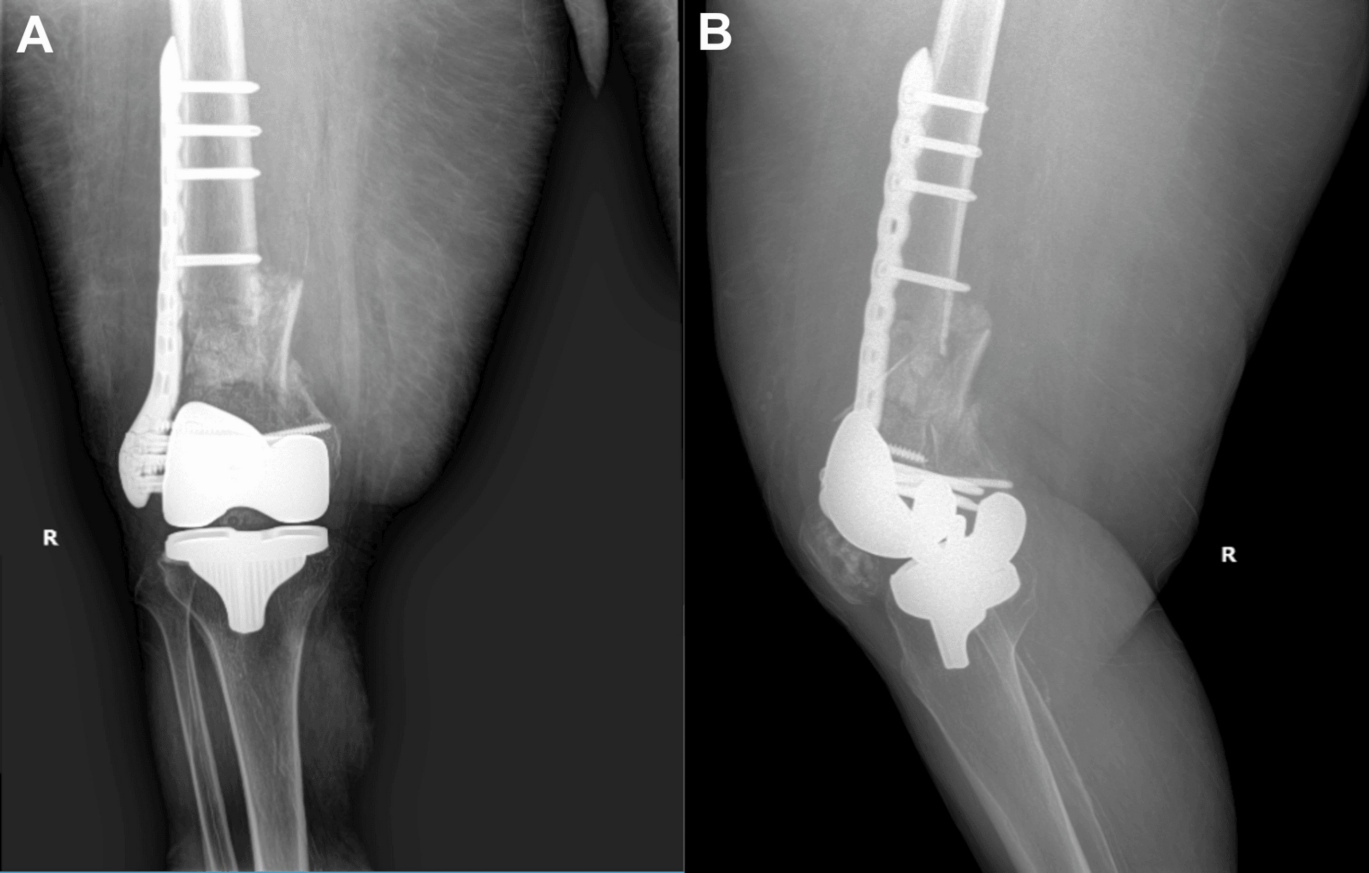
Radiographs obtained at four months postoperative following fixation of a periprosthetic distal femur fracture (A) Anteroposterior and (B) oblique radiographs of the right femur taken four months after open reduction and internal fixation of a comminuted periprosthetic distal femoral fracture proximal to a TKA implant. A lateral distal femoral locking plate was used for fixation. The TKA components remained well-aligned with no signs of loosening, and callus formation is evident.

Postoperative radiographs demonstrated stable fixation with appropriate alignment, length, and rotation. The patient commenced partial weight-bearing at six weeks post-surgery, progressing to full weight-bearing by 12 weeks. Postoperative serial radiographs seen in Figure 2 showed progressive callus formation, and the patient achieved pain-free ambulation by four months, with no signs of loosening or implant failure.

## Discussion

Distal femur replacement versus plate fixation

In managing distal femur fractures around TKA, the surgical team faced a critical decision between performing a distal femur replacement and utilizing the AxSOS 3 Ti Distal Lateral Femur Plating System. Each approach has specific advantages and limitations that must be carefully considered.

Distal femur replacement is often used in cases of severe comminution, nonunion, or bone loss, particularly in elderly patients with osteoporosis. It facilitates early mobilization and mitigates complications associated with prolonged immobilization, such as venous thromboembolism and stiffness [4]. However, distal femur replacement carries potential risks such as infection and the need for revision surgery [5]. Furthermore, it is more invasive and is generally reserved for cases where stable fixation cannot be achieved with less invasive techniques [6].

The AxSOS 3 Ti Distal Lateral Femur Plating System was selected in this case due to its ability to provide stable fixation while preserving the integrity of the existing knee prosthesis. Locking plates are particularly beneficial in osteoporotic bone, as they minimize the risks of nonunion and misalignment [7]. Additionally, the lateral approach minimizes soft tissue disruption, which facilitates quicker recovery and rehabilitation. The system's design allows for anatomical contouring and optimal screw placement, making it highly effective for fractures classified as AO/OTA 33A.

Rationale for plate fixation in this case

Several fixation strategies exist for managing periprosthetic distal femur fractures in patients with osteopenic bone, including dual plating, nail-plate combinations, and distal femur replacement. These enhanced fixation constructs may offer greater stability in cases with extensive comminution or poor bone stock. However, in the present case, the patient’s fracture pattern, preservation of condylar bone stock, and well-fixed prosthesis allowed for stable reconstruction using a single lateral locking plate. This approach avoided the additional surgical morbidity associated with more extensive reconstruction while providing sufficient fixation to support progressive weight-bearing and fracture healing.

The fracture's location and the patient's bone stock in the articular segment favored plate fixation over the more invasive distal femur replacement. This fracture pattern was amenable to open reduction and internal fixation using the plate. The surgical team opted to use the less invasive route when compared to distal femur reconstruction to achieve proper length, alignment, and rotation. Locking plates have demonstrated excellent outcomes in terms of biomechanical stability, fracture healing, and preservation of joint function, but they also have complications such as fixation failure due to metaphyseal comminution and nonunion [8]. The AxSOS 3 Ti system provided adequate fixation without the need for revision arthroplasty or prosthesis removal, reducing surgical morbidity and recovery time. 

## Conclusions

This case illustrates the effective management of a distal femur fracture around a TKA using the AxSOS 3 Ti Distal Lateral Femur Plating System. The choice of plate fixation allowed for stable fracture healing while preserving the prosthesis and minimizing surgical invasiveness. This approach is particularly suitable for patients with adequate bone stock in the condylar region and intact prostheses. 
